# Rumen acidosis in ruminants: a review of the effects of high-concentrate diets and the potential modulatory role of rumen foam

**DOI:** 10.3389/fvets.2025.1595615

**Published:** 2025-05-27

**Authors:** Jinju Mao, Lizhi Wang

**Affiliations:** Animal Nutrition Institute, Sichuan Agricultural University, Chengdu, China

**Keywords:** rumen acidosis, pH, saliva, volatile fatty acids, carbon dioxide, rumen foam

## Abstract

This review delves into the intricate processes by which high concentrate diets (HCD) in ruminants trigger rumen acidosis, with particular attention to the initiating factors of the condition and the pivotal role of rumen foams in its progression. High concentrate diets lead to an excessive accumulation of acids within the rumen, creating a favorable environment for the formation of rumen foam. This foam exacerbates the severity of rumen acidosis, making it a more challenging condition to manage. Additionally, HCD significantly diminishes salivary secretion, which not only increases the viscosity of rumen contents but also hampers the absorption of volatile fatty acids and the release of carbon dioxide (CO₂). Moreover, the review highlights a previously underexplored mechanism: the build-up of CO₂ may play a crucial role in the pathogenesis of rumen acidosis. This oversight could have significant implications for understanding the onset and advancement of the condition. In essence, this paper seeks to establish a robust scientific framework to optimize ruminant nutrition and production practices, ultimately ensuring the health and well-being of these animals.

## 1 Introduction

In China, the scarcity of high-quality roughage has necessitated the use of substantial amounts of grain-rich concentrate feeds in ruminant breeding. For example, large quantities of cereals such as maize and wheat, as well as soya bean meal and rapeseed meal, are used as concentrate ingredients, which can total more than 60%. Farmers adopt this strategy to meet nutritional requirements and maximize returns. However, this feeding pattern, characterized by high concentrations of fermented starch, leads to the rapid production of volatile fatty acids (VFAs) in the rumen. The excess VFAs lower the pH, potentially causing disruptions in rumen microbial balance (1). Consequently, prolonged consumption of high-concentrate diets (HCD) can trigger nutritional metabolic disorders, with rumen acidosis being a prevalent issue that significantly affects ruminant productivity (2–4). Despite the widespread occurrence of rumen acidosis, the precise mechanisms remain elusive, sparking considerable debate among researchers. To address this gap, this article aims to provide a comprehensive review of the initiating factors of rumen acidosis within the context of HCD feeding. By establishing a robust theoretical foundation, we hope to offer valuable insights for the nutritional management of rumen acidosis in ruminant farming.

## 2 Overview of rumen acidosis

Rumen acidosis is a metabolic nutritional disorder that arises from the feeding of HCD, which increase the susceptibility of the rumen to fermentation and result in excessive acidic accumulation. This leads to a decrease in rumen pH, leading to significant pH depression and potentially triggering inflammatory responses. The disorder can severely affect the growth and development of ruminants during breeding, resulting in considerable economic losses for farmers (5, 6). Currently, ruminal acidosis is classified into acute and subacute forms based on the clinical signs and pH variations. Acute rumen acidosis is characterized by a rumen pH below 5.0, lactate levels surpassing 50 mM/L, and VFAs concentrations below 100 mM/L (7). While acute rumen acidosis can cause direct mortality in ruminants, it occurs less frequently (8). Subacute rumen acidosis (SARA) is not clearly defined, but it is generally accepted that SARA occurs if rumen pH falls below 5.6 for more than 3 h per day or below 5.8 for more than 5.4 h per day (9–12). Affected cattle and sheep often display symptoms such as reduced appetite, decreased feed intake, lameness, diarrhea (often containing undigested grain and/or gas in the feces), and dairy cows may suffer a significant decrease in milk fat content, hoof inflammation, and liver abscesses. Although less severe than the acute form, subacute ruminal acidosis (SARA) causes more damage related to production because of its higher incidence rate (8). Previous studies have shown that in the North American dairy industry, an annual economic loss of 5–10 billion US dollars is attributed to SARA (13). In Europe, the incidence rate in mid-lactation dairy cows can be as high as 26%, and in early lactation, it can reach 19% (14). In China, the incidence of rumen acidosis in the beef and dairy cattle industry ranges from 9 to 26%, greatly impacting farm profitability (15).

## 3 The development process of rumen acidosis

**Figure 1:**
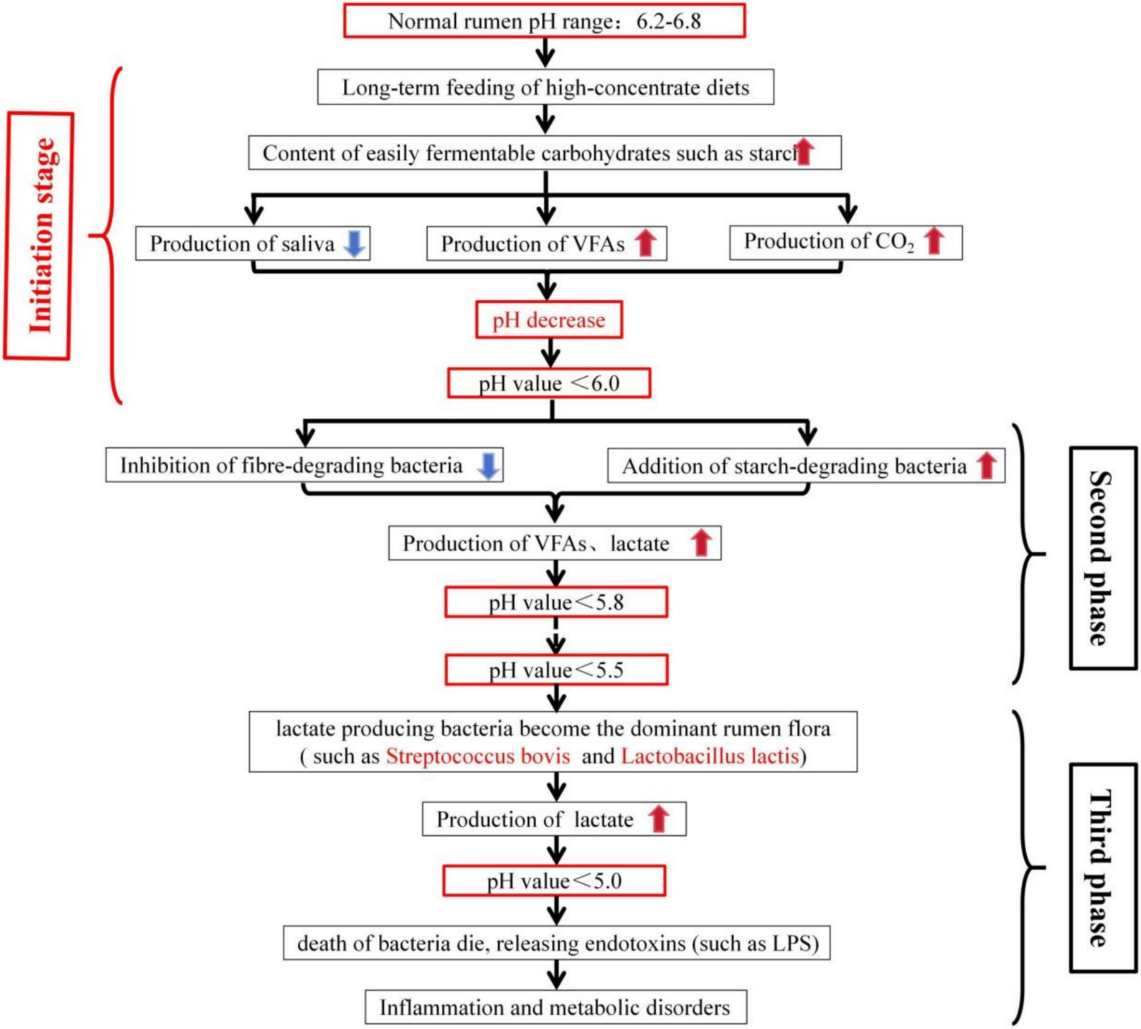
Sketch of the development of rumen acidosis.

At present, the prevalent descriptions of rumen acidosis are generally along these lines: when ruminants are fed substantial quantities of HCD, the high starch content prompts rapid fermentation by rumen microbes, yielding volatile fatty acids (VFAs) and lactate, leading to a decrease in pH. This pH disequilibrium then precipitates the occurrence of ruminal acidosis. In correlation with the analysis by Valente et al. (16), the progression of ruminal acidosis is encapsulated in Figure 1.

### 3.1 Initiation phase

During the early phase, after ingesting large quantities of HCD, ruminants show a decline in both the frequency and duration of their daily rumination. The phenomenon occurs due to the lower fiber content and higher starch levels in HCD. This results in reduced saliva production, which decreases the supply of buffers needed to neutralize acids in the rumen, thereby impairing the rumen’s buffering capacity (17, 18). Moreover, the fermentation substrates in HCD are mainly composed of soluble carbohydrates, which leads to the production of substantial amounts of VFAs, CO_2_, and other acids that start to accumulate. Consequently, the rumen pH experiences its first decline, dropping below the normal range of 6.2–6.8 to below 6.0. A stable and balanced ecosystem is maintained in the rumen, with microbial communities thriving within an optimal pH range, typically between 6.0 and 7.0. Under normal pH conditions, the rumen microbial community is both diverse and stable, encompassing fiber-degrading bacteria (such as *Fibrobacter succinogenes*, *Ruminococcus flavefaciens*), starch-degrading bacteria (such as *Streptococcus bovis*, *Ruminobacter amylophilus*), and methanogens (such as *Methanobrevibacter ruminantium*). However, the microbial community in the rumen undergoes significant changes when the pH falls due to the intake of HCD. This alteration in the composition of the microbial community disturbs the rumen’s equilibrium, leading to further acid build-up and metabolic disturbances (7). Thus, this phase marks the onset of rumen acidosis development.

### 3.2 Second phase

After the rumen pH falls below 6.0, the environment for rumen microorganisms changes, resulting in a decrease in the growth of fiber-degrading bacteria that are highly sensitive to pH fluctuations. This sensitivity is evident in a reduction of both their activity and numbers (19). In contrast, the population of amylolytic bacteria increases, particularly the lactic acid-producing species like *Streptococcus bovis* and Lactobacillus spp., which flourish at lower pH levels and quickly ferment starch. The increased fermentative action raises the production of VFAs, which in turn causes the rumen pH to drop further, to below 5.8 (17, 20). In this scenario, the proliferation of lactic acid-producing bacteria (e.g., *Streptococcus bovis*) leads to enhanced lactic acid production, causing the rumen pH to decrease further to below 5.6. This dip creates a markedly acidic rumen environment, disrupting its normal function (7, 17), marking this stage as the second phase of rumen acidosis.

### 3.3 Third phase

When the rumen pH falls below 5.6, the growth and reproductive abilities of most rumen microorganisms are compromised due to the overly acidic environment. This heightened acidity promotes the proliferation of lactate-producing bacteria, such as *Streptococcus bovis* and *Lactobacillus lactis*, within the rumen flora. This leads to the excessive production and accumulation of lactate. As a result, the rumen pH drops rapidly to below 5.0 (7, 20). Under such conditions, many bacteria die and release endotoxins such as lipopolysaccharides (LPS), which can further impair rumen health and potentially triggering systemic inflammation (21, 22). This stage is considered the third phase of rumen acidosis.

## 4 Causes of initiation of rumen acidosis

Upon examining the definition of rumen acidosis and the described development process, it is evident that pH serves as a crucial foundation and criterion for the assessment of rumen acidosis by the majority of scholars. The pH value characterizes the acidity or alkalinity of a solution (23). It reflects the hydrogen ion (H^+^) activity in the solution. As the solution becomes more acidic, the H^+^ concentration increases. Normally, the pH of rumen fluid falls within the range of 6.2 to 6.8. During the initial phase of rumen acidosis development, the rumen pH drops from the normal range to below 6.0. The primary potential triggers for this decrease in rumen pH are as follows.

### 4.1 Decreased saliva production

In ruminant digestion, saliva plays a crucial role as one of the primary fluids that enter the rumen. After ingesting roughage, ruminants such as cattle can produce a daily salivary output exceeding 150 liters (24), whereas in sheep, it generally ranges between 1.2 and 10.2 liters per day (25). The concentration of dry matter in ruminant saliva is approximately 1.0–1.4 mg/mL, consisting mainly of inorganic substances like sodium, bicarbonate, potassium, phosphate, and a smaller amount of chloride (26, 27). Due to high levels of bicarbonate and phosphate, the saliva is alkaline (28). J. T. Reid et al. (29) documented the salivary pH for various ruminant species: buffaloes at a pH of 8.8; sheep between 8.12 and 8.32; goats from 8.2 to 8.8; calves with a pH of 8.1–8.23; and cattle ranging from 8.55 to 8.90, averaging 8.8. The alkaline nature of saliva helps to neutralize acids and, combined with its lubricating effects on the food bolus, aids in chewing and swallowing. This action helps to minimize the accumulation of VFAs and maintains the rumen’s pH balance (30). It is estimated that a cow’s saliva can neutralize about 30 to 40% of the VFAs produced in the rumen (24). Additionally, research indicates that the 24-h salivary output of an adult ruminant contains 300–350 grams of sodium carbonate, which can neutralize 56.6–65.0 liters of a 0.1 mol/L hydrochloric acid solution (29). Moreover, the alkalinity of substances is commonly assessed based on the pKa of their conjugate acid, with higher pKa values denoting greater alkalinity. In ruminant saliva, the pKa values for bicarbonate and phosphate are 10.25 and 12.67, respectively, emphasizing their significant acid-neutralizing ability.

Research suggests that in ruminants, there is a direct relationship between the frequency of chewing and the production of saliva (31). Conversely, feeding ruminants diets high in concentrates reduces fiber content and results in smaller feed particle sizes, leading to fewer chewing events and decreased salivary secretion, thus diminishing their capacity to counteract rumen acidity (31–33). K. Jacques and colleagues (34) found that the proportion of roughage in the diet influences salivary secretion, noting an increase in salivary outflow when the hay content in the diet was increased from 50 to 90%, even with a constant intake of ruminal fluid. An increase in hay proportion or intake directly enhanced salivary flow. Furthermore, G.E. Chibisa (35) and his team observed that cows on low-fiber, high-grain diets consumed their food more rapidly, exhibited significantly less salivary secretion during meals, and secreted less saliva at rest. Ezequias Castillo-Lopez (36) and his team discovered that prolonged consumption of HCD led to a decrease in rumen pH, an effect worsened by the reduction in feed-induced salivation and chewing activity. Consequently, the reduced fiber content and increased concentrate in HCD decrease the stimulation for salivary secretion, leading ruminants to chew and ruminate less. This results in a further reduction in salivary secretion, depleting salivary buffer components like bicarbonate, which are vital for neutralizing acids (18, 35, 37).

### 4.2 Accumulation of VFAs

VFAs are critical end-products of rumen fermentation in ruminants, primarily synthesized through microbial degradation of carbohydrates like fiber, starch, and sugar (24). The main VFAs - acetic, propionic, and butyric acids - make up about 40–70%, 15–40%, and 10–20% of the total ruminal VFAs, respectively (38). Rumen acid buffering relies on bicarbonate from saliva, epithelial VFA absorption (which removes 50–80% of acid, with each VFA mole eliminating ~0.5 H + moles (39)), and proton efflux. Extra acid is neutralized by saliva or sent to the small intestine (24, 40). When ruminants eat HCD, the non-structural carbs boost microbial fermentation, causing VFA production to exceed clearance and leading to accumulation. (7, 41). E. J. Carroll et al. found that VFA production rates go in this order: grain-fed > hay-fed > pastured animals. (42). As weak acids with similar pKa values (~4.80), VFAs mostly dissociate in the rumen, lowering pH (43). Henderson-Hasselbalch calculations (Equation 1) suggest that over 95% of acetate, propionate, and butyrate are present in dissociated form at a pH of 6.0 (43). After the ingestion of HCD, rapid VFAs production initially decreases rumen pH. However, as H^+^ ion concentration reaches equilibrium dictated by the pKa values, the dissociation equilibrium stabilizes, limiting further pH decline. This buffering effect generally restricts the reduction of rumen pH to approximately 5.8 during prolonged VFAs accumulation.


(1) $$\mathrm{pH}={\mathrm{pK}}_{a}+\mathrm{lg}\frac{\left[\text{Proton acceptors}\right]}{\left[\text{Proton donors}\right]}$$

Ramos et al. (44) demonstrated that shifting the dietary concentrate-to-forage ratio from 2:8 to 8:2 elevated total VFAs concentration from 92.39 to 154.59 mmol/L. During sustained HCD feeding, VFAs levels stabilized around 150 mmol/L, concurrent with a marked decline in rumen pH from 6.87 to 5.58. Similarly, Chibisa et al. (35) observed significantly higher total VFAs (118 mM vs. 95 mM), osmolality, and molar proportions of propionate/isovalerate in cattle fed low-fiber, high-grain (LF) diets compared to high-fiber (HF) diets. Despite equivalent VFAs absorption rates by rumen epithelium between dietary groups, LF-fed cattle exhibited exacerbated ruminal acidosis indices. This suggests that accelerated VFAs production rates under LF conditions exceeded clearance capacity, leading to VFAs accumulation and subsequent pH decline (35). Shen et al. (45) further corroborated these findings, reporting that increased dietary concentrate proportion induced elevated rumen osmolality alongside significant rises in propionate, butyrate, and total VFAs concentrations. Notably, their study revealed a positive correlation between rumen osmolality and total VFAs (LC: 90.34 mM; MC: 101.96 mM; HC: 113.95 mM). Elevated osmolality impaired paracellular resistance, suppressed apical Na^+^-H^+^ exchange activity, and compromised epithelial integrity. Critically, osmotic overload was shown to inhibit VFAs absorption, exacerbating acid retention (45). In summary, these studies support the hypothesis that HCD-induced microbial fermentation rapidly generates VFAs exceeding epithelial absorption capacity. This imbalance results in ruminal VFAs accumulation and subsequent acidotic pH depression (46).

### 4.3 Accumulation of CO_2_

Current understanding of ruminal acidosis pathogenesis predominantly attributes pH reduction to VFA or lactic acid accumulation, while existing studies largely neglect the contributory role of carbon dioxide (CO₂) (47). In the rumen ecosystem, CO₂ primarily originates from microbial decarboxylation of VFAs (e.g., acetate, propionate, butyrate) during feed fermentation. HCD, rich in rapidly fermentable carbohydrates, intensify microbial activity, thereby amplifying CO₂ production. Notably, elevated ruminal dissolved CO₂ (dCO₂) concentrations may independently drive pH depression through acid–base perturbations (47). CO₂ exhibits high aqueous solubility, existing in three equilibrium states within ruminal fluid: Hydrated form (dCO₂: CO₂·H₂O), Acidic species (carbonic acid, H₂CO₃) and Conjugate base (bicarbonate, HCO₃^−^) (Equation 2) (47). Mechanistically, dCO₂ forms labile associations with hydronium ions (H₃O^+^). During active fermentation, excessive CO₂ generation increases H₃O^+^ activity through dCO₂ protonation, thereby acidifying ruminal fluid (48). This CO₂-mediated proton release establishes an underappreciated pathway for pH reduction in HCD-induced acidosis.


(2) $$\begin{array}{l} {\mathrm{CO}}_{2}\underset{1}{\Leftrightarrow }\mathrm{dC}O_{2}(CO_{2}+nH_{3}O^{+})\underset{2}{\Leftrightarrow }H_{2}CO_{3}\\ +nH_{3}O^{+}\underset{3}{\Leftrightarrow }\mathrm{HC}O_{3}^{-}+nH_{3}O^{+} \end{array}$$

In addition, rumen pH can also be better explained by the balance between dCO_2_ and HCO_3_^−^ concentrations (Equation 3). Equation 3 illustrates that HCO_3_^−^ comprises the principal form of CO_2_ at pH levels exceeding the equilibrium point (pKa = 6.37). For instance, within blood (pH 7.4), CO_2_ is largely present as HCO_3_^−^ (88%), with a minimal quantity (5%) existing as dCO_2_ (49). Conversely, during the progression of rumen fermentation, dCO_2_ becomes the predominant CO_2_ form in the rumen fluid, causing a drop in pH. HCD are typically rich in rumen-degradable starch (RDS), enhancing CO_2_ production and increasing the viscosity of rumen fluid (47). Moreover, HCD diets exhibit a reduced fraction of physically effective neutral digestible fiber (pe NDF) (50), leading to diminished rumen chyme particle sizes and elevated rumen gas content (51). These alterations in the physicochemical characteristics might decrease CO_2_ volatility, elevate CO_2_ retention, and augment the concentration of dCO_2_, potentially influencing rumen pH. Laporte-Uribe’s (48) study have demonstrated that rumen CO_2_ retention is linked with substantial dCO_2_ levels and modifications in HCO_3_^−^ activity, rumen fluid viscosity, and surface tension, which are evident during the CO_2_ retention phase in SARA.


(3) $$\mathrm{pH}={\mathrm{pK}}_{a}+\log\frac{\left[{{\mathrm{HCO}}_{3}}^{-}\right]}{\left[{\mathrm{dCO}}_{2}\right]}$$

## 5 Causes of rumen accumulation of VFAs and CO_2_

The analysis of the triggers presented earlier reveals that the build-up of VFAs and CO_2_ plays a pivotal role in the onset of rumen pH decline. This segment delves into the factors contributing to this accumulation, aiming to enhance the comprehension of the processes leading to the drop in rumen pH.

### 5.1 Excessive production rate

The elevated levels of non-structural carbohydrates in high concentrate diets enhance the rate of rumen microbial fermentation, resulting in a considerable production of VFAs and CO_2_. The pace of acid production surpasses the rate of absorption, leading to the accumulation of acid within the rumen (52, 53). This phenomenon is commonly attributed by many scholars to the cause of ruminal acidosis. Nevertheless, due to concurrent processes of VFAs production and absorption, along with CO_2_ production and emission in the rumen, it is challenging to ascertain precise data on these rates. Some scholars’ studies could demonstrate that feeding HCD resulted in faster rates of VFAs production and higher yields. For example, some scholars (42) have indirectly gaged VFAs production rates through *in vitro* fermentation experiments, discovering that cereal-fed animals exhibit the highest VFAs production, while hay-fed animals have a moderate rate, and pasture-fed animals display a slightly lower rate. The lowest VFAs production was observed in pasture-fed animals, partially affirming the conjecture that VFAs production is excessively rapid with HCD feeding. However, there is little direct evidence on the rate of uptake of VFAs in the rumen, and the majority of studies have only assessed the rumen VFAs concentration post high concentrate diet consumption, exemplifying an increase that may lead to VFAs accumulation. Whether this increase results from a significantly higher production rate than absorption rate awaits confirmation from more direct studies.

### 5.2 Presence of rumen foam

It has been noted that the ingestion of HCD may result in the formation of numerous stable foams within the rumen, leading to foam-type flatulence (54). The causes of rumen foam associated with HCD intake include diminished salivary output, augmented rumen fluid viscosity, decreased surface tension, and an elevated presence of polysaccharides and proteins acting as ‘foaming agents’ in the rumen content, promoting bubble formation and stability. Research indicates that animals fed HCD exhibit a marked increase in foam stability, production, and rumen fluid viscosity compared to those on low concentrate diets (55), predisposing them to the development of extensive rumen foams, thereby precipitating flatulence. This can be due to several factors. High levels of carbohydrates, particularly non-structural carbohydrates such as starch and sugar, are commonly associated with increased foam production. These carbohydrates also lead to rapid fermentation by rumen microorganisms, producing gasses such as carbon dioxide and methane, which can lead to foam formation. Additionally, certain proteins and peptides in feeds may act as surfactants, stabilizing foam. These foams might hinder the rumen epithelium’s absorption of VFAs, leading to VFAs accumulation and subsequent reduction in rumen pH. Qy and colleagues (56) observed that incorporating an antifoam agent, dimethylsiloxane oil, into HCD diets effectively dissipated rumen foams, lowered VFAs concentrations in rumen fluid, and elevated rumen pH. Zhang (57) found a close link between the amount of polysaccharides in rumen fluid and its viscosity, foaming ability, and foam stability. This points to a key factor in rumen foam formation. Later studies showed that non-starch polysaccharidases in the diet can greatly cut down polysaccharide levels. This in turn reduces foam traits such as bubble stability, formation ability, and viscosity. Notably, these enzymes increased rumen pH and decreased the levels of acetic and propionic acids. It was postulated that rumen foam could obstruct VFAs contact with the rumen wall, diminishing VFAs absorption and causing rumen VFAs build-up. During HCD consumption, rumen liquid viscosity and surface tension are reduced, conditions favorable for foam generation. Foam presence may also inhibit rumen motility, disrupting normal gas flow and leading to CO_2_ accumulation. Moreover, abundant rumen foam raises internal pressure, enhancing CO_2_ solubility and dCO_2_ concentration, contributing to pH decline. Further studies are needed to substantiate these theories. Additionally, saliva contains minor foam inhibitors (58), and HCD-induced reduction in saliva reduces the availability of these inhibitors, failing to prevent foam formation. The cumulative effect of foam formation due to HCD, with its high starch and soluble carbohydrate content, creates an environment fostering stable foam presence, leading to VFAs and dCO_2_ accumulation, a drop in rumen pH, and the onset of rumen acidosis.

## 6 Conclusion and outlook

In conclusion, this review highlights that rumen acidosis in ruminants is mainly caused by acid accumulation within the rumen, with rumen foam possibly making it worse. Chronic feeding of HCD reduces saliva production and raises digesta viscosity, promoting foam formation. This foam hinders VFA absorption and affects CO_2_ release, lowering rumen pH and increasing acidosis risk. To prevent this, nutritional interventions should focus on adjusting dietary composition to minimize acidosis risk, enhancing salivary buffering capacity, and reducing rumen foam formation. Future research should investigate the efficacy of dietary additives and feed enzymes in supporting rumen health, as well as the development of monitoring technologies for early detection of acidosis. By prioritizing these strategies, the industry can optimize ruminant nutrition and production efficiency, ensuring animal welfare and promoting environmental sustainability.
